# Natural killer cell-based cancer immunotherapy: from basics to clinical trials

**DOI:** 10.1186/s40164-024-00561-z

**Published:** 2024-10-16

**Authors:** Yinghong Shi, Donglin Hao, Hui Qian, Zhimin Tao

**Affiliations:** 1 Wujin Institute of Molecular Diagnostics and Precision Cancer Medicine of Jiangsu University, Wujin Hospital Affiliated With Jiangsu University, Changzhou, 213017 Jiangsu China; 2 Jiangsu Province Key Laboratory of Medical Science and Laboratory Medicine, School of Medicine, Jiangsu University, Zhenjiang, 212013 Jiangsu China; 3 Department of Emergency Medicine, Affiliated Hospital of Jiangsu University, Zhenjiang, 212001 Jiangsu China

**Keywords:** Natural killer cells, Immunotherapy, Tumor microenvironment, Chimeric antigen receptor, Clinical trial

## Abstract

Cellular immunotherapy exploits the capacity of the human immune system in self-protection and surveillance to achieve the anti-tumor effects. Natural killer (NK) cells are lymphocytes of innate immune system and they display a unique inherent ability to identify and eliminate tumor cells. In this review, we first introduce the basic characteristics of NK cells in the physiological and pathological milieus, followed by a discussion of their effector function and immunosuppression in the tumor microenvironment. Clinical strategies and reports regarding NK cellular therapy are analyzed in the context of tumor treatment, especially against solid tumors. Given the widely studied T-cell therapy in the recent years, particularly the chimeric antigen receptor (CAR) T-cell therapy, we compare the technical features of NK- and T-cell based tumor therapies at the clinical front. Finally, the technical challenges and potential solutions for both T and NK cell-based immunotherapies in treating tumor malignancies are delineated. By overviewing its clinical applications, we envision the NK-cell based immunotherapy as an up-and-comer in cancer therapeutics.

## Introduction

The research history of immune system fighting against cancer may trace back to the late nineteenth century when William Coley discovered cases of spontaneous cancer regression following infection [1]. Nowadays, immunotherapy has shown remarkable success in treating a variety of cancers. The approaches comprise of cancer vaccines, checkpoint inhibition and chimeric antigen receptor (CAR) T cell therapies, especially the last two of which brought cancer immunotherapy into a new era [2]. The clinical outcomes of CAR-T cell therapies in treating patients with hematological malignancies have been very inspiring, which encourages research efforts to explore the clinical use of other types of immune cells, such as natural killer (NK) cells and macrophages [3], with hopes to circumvent some limitations of CAR-T cells [4].

NK cells were first characterized in 1976 as a class of lymphoid cells that differed from T cells and B cells [5]. Initially NK cells were believed to only possess occasional cytotoxic activity, but they are now considered a critical and indispensable entity against malignant tumors without prior sensitization and independent of major histocompatibility (MHC) restriction [6]. NK cells have special activating and inhibitory receptors on their surfaces. Upon recognizing and binding with corresponding ligands, NK cells function under the instruction by the input of activating-to-inhibitory signal [7]. They not only directly lyse tumor cells, but also mediate antibody-dependent cellular cytotoxicity (ADCC) and regulate T cell function to eliminate tumor cells [8].

Due to the intrinsic ability to eradicate tumor cells, NK cell-based cancer therapies have attracted great attention. Early clinical trials have proved the overall safety of autologous NK cell infusion, albeit only a limited antitumor effect was observed [9, 10]. The main reason for this limited therapeutic effect was that the self-HLA molecules on the tumor cells led to a suppression of autologous NK cells by killer cell immunoglobulin-like receptors (KIRs). To overcome this suppression, allogeneic or haploidentical NK cells lacking KIR ligands were used to achieve remarkable and safe antitumor effects in vivo [11]*.*

In this review, we introduce the basic characteristics of NK cells, summarize the role of NK cells against cancers, and delineate possible mechanisms of their functional impairment in cancers. Then, NK cell-based immunotherapy strategies are highlighted with exemplified clinical trials to analyze the achievements and challenges in cancer treatment. Since NK cell infusion has been one of the most popular immunotherapies in the recent years, preclinical studies and clinical trials of NK cell infusion in malignant tumors, especially solid tumors, are discussed below. Finally, we compare the features of NK- and T-cell based tumor therapies in the clinical settings, followed by analysis of the clinical challenges and current solutions in CAR related cell therapies.

## Development and classification of NK cells

NK cells have been recognized as typical innate immune cells, especially a member of group 1 innate lymphoid cells (ILCs) [6]. ILCs represent a heterogeneous group of cells that lack genetically rearranged antigen receptors [12]. The subsets of ILCs are classified with different capacities to produce cytokines and transcription factors required for development and function. NK cells are characterized by their capacity to produce large amounts of interferon-γ (IFN-γ) and their functional reliance on the transcription factor T-bet, albeit not strictly dependent on T-bet for their development [13].

Human NK cells derived from multipotent CD34^+^ hematopoietic progenitors predominantly initiate in the bone marrow. The hematopoietic progenitors first develop into common lymphoid progenitors. Then, they differentiate into a group of heterogeneous cells including pre-NK precursors and other innate lymphoid precursors [14]. Next, NK precursors, which are derived from pre-NK precursors, express the interleukin-15 receptor complex (IL-15R), which consists of β and γ subunits [15]. At this stage, the cells also begin to produce representative surface marker CD56. Due to the different expression levels of CD16, the NK cells can be divided into two subgroups: CD56^bri^CD16^−/low^ and CD56^dim^CD16^+^ [14, 16].

The CD56^dim^CD16^+^ NK cells are fully mature, which constitute ~ 90–95% of the NK cell population in peripheral blood and predominantly mediate cytolytic responses [17].The other CD56^bri^CD16^−/low^ cells are relatively immature, which make up ~ 5–10% of total NK cells and produce a number of cytokines, including IFN-γ, TNF-α, and granulocyte–macrophage colony-stimulating factor (GM-CSF). While playing a weak role in cytolytic responses, these cells are critical in developing type-1 T cell responses for the role of IFN-γ in antigen presentation [18]. NK cells therefore not only act as killers, but also have immunoregulatory functions. Studies have shown that these two NK cell subsets are separate lineages; however, immature CD56^bri^CD16^−/low^ cells are also thought to be precursors of CD56^dim^CD16^+^ mature NK cells, and can reach a terminal differentiation into CD56^dim^CD16^+^ NK cells after exposure to IL-2 or/and IL-15 [19]. In contrast to T cells, the maturation of NK cells takes place in the bone marrow and secondary lymphoid tissues, but not in the thymus [15]. NK cells circulate and widely distribute throughout the body under the instruction of homing molecules. CD56^bri^ CD16^−/low^ NK cells express the chemokine receptor CCR7 and L-selectin, which drive their migration to secondary lymphoid organs. In contrast, CD56^dim^ CD16^+^ NK cells display a high density of CX3CR1 and CXCR1, which direct them into peripheral tissues [20].

Recently, mounting evidence confirmed the presence of tissue-resident NK cells (trNKs). The above described conventional NK cells (cNKs) are able to leave the vasculature and migrate to a specific location to exert their effects [21], while trNKs are distinct from these cNKs in the expression of cell surface molecules and transcription factors [22]. For example, renal resident NK cells were CD49a^+^CD49b^−^ and moderately reduced in T-bet-deficient mice, but increased in NFIL3-deficient mice [23]. In contrast, salivary glands-derived CD49a^+^CD49b^+^ NK cells required neither T-bet nor NFIL3 for development, as TGF-β plays a vital role in shaping the characteristics of salivary gland trNKs [24]. These results demonstrated that different tissue-derived trNKs have unique tissue-specific features, possibly shaped by tissue microenvironment [25, 26].

Research findings discovered the immunological memory in NK cells besides their role in innate immunity [27]. There are two major types of stimuli to generate memory-like NK cells (MLNK). First, like T cells or B cells, NK cells can exert immunological memory after being re-stimulated with haptens or viruses, resulting in antigen-specific memory NK cells [28]. Second, cytokine-induced memory-like (CIML) NK cells are a group of long-lasting receptor dependent cells that differ from antigen-specific NK cells. Incubation of NK cells with interleukin IL-12, IL-15, and IL-18 could produce CIML NK cells with enhanced responses to cytokine or activating receptor restimulation for weeks to months [29].

Other types of specific NK cells, such as HLA-DR^+^NK cells, combine phenotypic characteristics of both NK cells and dendritic cells. They are able to present antigens to T cells and induce their activation and proliferation [30]. Regulatory NK cells (CD27^+^CD11b^+/−^) are an immunomodulatory subset of NK cells with reduced antitumor cytotoxicity and increased secretion of immunosuppressive cytokine IL-10 and TGF-β [31, 32]. NK cells acquire ‘helper’ activity in the induction of dendritic cells (DCs) maturation into stable type-I polarized DCs with proper stimulation. In addition to tumor cell-derived signal, one soluble factor, IL-18, may synergize with type-1 IFNs or IL-2 in the induction of ‘helper’ NK cells [33]. Taken together, all phenotypes of NK cells fulfill their individual and specific functions to maintain body homeostasis.

## The role of NK cells in cancers

### NK cells as the anti-tumor effectors

Being important effectors in innate immunity, NK cells can function as killers to release inflammatory cytokines without pre-sensitization. They not only destroy tumor cells directly, but also mediate ADCC and regulate the function of T cells [34]. These functions depend on inhibitory and activating signals emitted by receptors expressed on their cell surface. Inhibitory receptors have the task of recognizing self-MHC class I ligands on healthy cells, preventing the activation of NK cells and protecting healthy cells from being dismantled [35]. The most important inhibitory receptors, the KIRs of KIR3DL1 and KIR2DL3/2DL1, lyse target cells that have lost (or express only small amounts of) MHC class I molecules [36]. The loss of expression of surface MHC class I molecules, frequently caused by viral infection or malignant transformation, results in ‘missing-self recognition’ by NK cells [37]. CD94 in combination with NK group 2 (NKG2) family, assembles CD94-NKG2 system, where CD94 forms a disulfide-linked heterodimer with the inhibitory NKG2A receptor or with the activating NKG2C receptor. Unlike KIRs, the CD94-NKG2 receptors are invariant and recognize the non-polymorphic MHC class I ligand, human leukocyte antigen-E (HLA-E) [34, 38]. Additionally, NK cells also express other non-MHC dependent inhibitory receptors, including killer lectin-like receptor G1, CD96, TIGIT, and LAG3 [21]. Programmed cell death 1 (known as PD-1) and other immune checkpoint receptors may also inhibit NK cell activation, particularly in the context of viral infections or cancers [36].

**Fig. 1:**
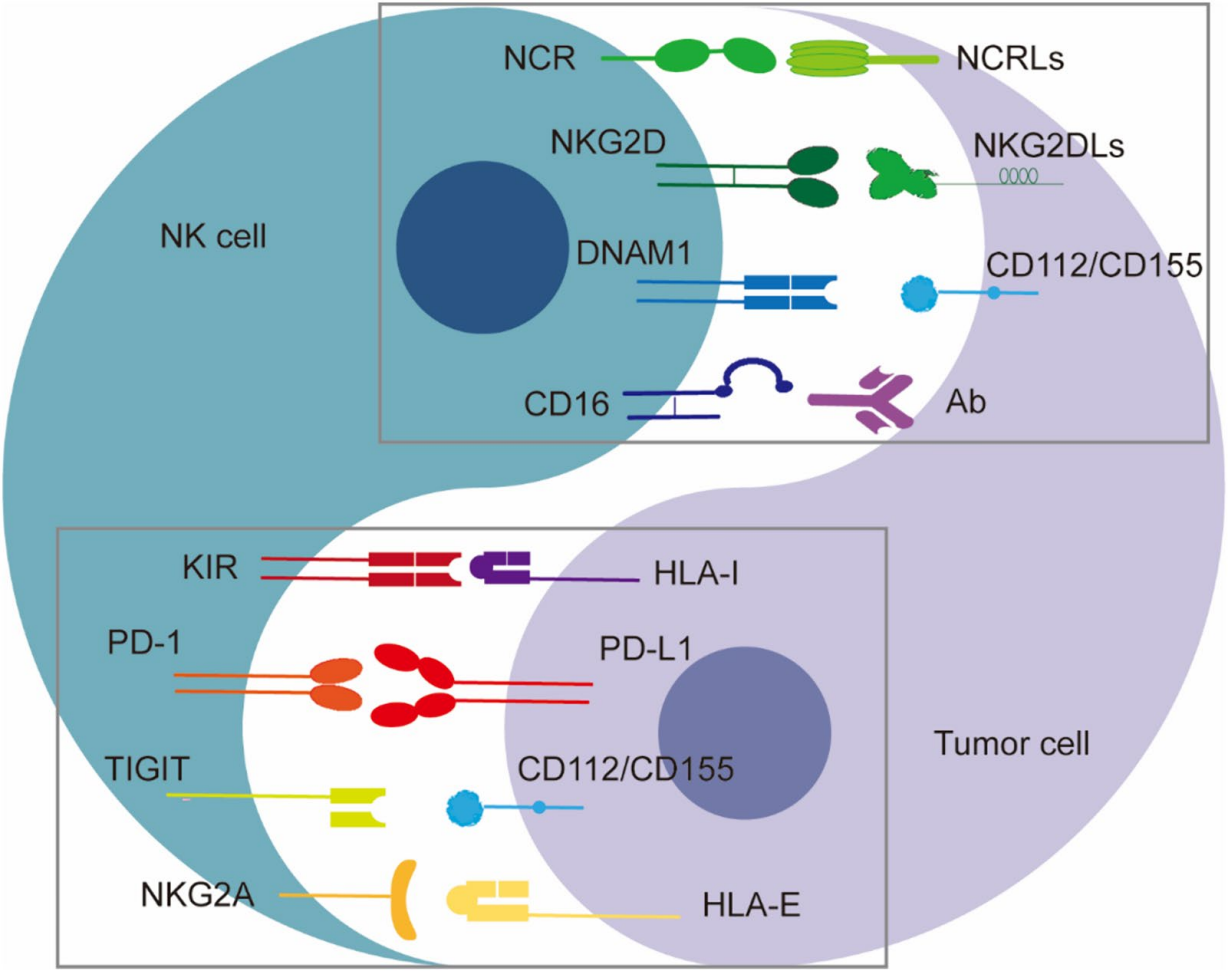
Schematic representation of known major NK cell receptors and their corresponding tumor cell ligands. Mature human NK cells express a range of germline-encoded activating and inhibitory receptors that bind specific ligands either constitutively expressed or induced on tumor cells during transformation. The expression levels of tumor ligands and NK cell receptors determine whether an NK cell will kill a tumor cell. The upper box shows matched activating receptors and ligands; the lower box shows matched inhibitory receptors and ligands

Activating receptors recognize inducible ligands expressed by stressed cells (including tumor cells and virus-infected cells). NKG2 member D (NKG2D), DNAX accessory molecule 1 (DNAM1), the natural cytotoxicity receptors (NCRs) including NKp30, NKp44, and NKp46, and immunoglobulin G Fc receptor FcγRIII (known as CD16), are well-characterized activating receptors [39]. NKG2D recognizes the stress-inducible MHC class I chain-related A and B genes (MICA/B) and UL16 binding proteins (ULBP) [40]. DNAM1 can bind to CD155 and Nectin-2 (CD112), two members of the nectin family present on most tumor cells. NKp30, NKp46 and NKp44 paired with B7-H6, influenza viral hemagglutinin and complement factor P and NKp44L, respectively [41]. Additionally, KIR2DS and KIR3DS, characterized with a short intracytoplasmic tail, are activating receptors of the KIR family, binding MHC class I molecules like inhibitory KIRs that bear a long intracellular tail [21]. Well known NK cell receptors and their corresponding tumor cell ligands are shown in Fig. 1.

Upon a positive signal delivered by tumor via interactions with NK cell receptors, NK cells activate and directly destroy tumors through degranulation, a process induced by ADCC [42]. The released granules contain perforin and granzyme. Perforin, as the name implies, perforates the target cell membrane, causing osmotic lysis, whereas granzyme induces caspase-dependent apoptosis of target cells [15]. CD16 is the key receptor that is engaged in ADCC recognizing immunoglobulin-opsonized cells [43]. It induces phosphorylation of the immunoreceptor tyrosine-based activation motif domains of the high-affinity IgE receptor (FcεRIγ) and CD3ζ in NK cells, and initiates a signaling cascade that ultimately results in killing of the antibody-coated cell [44]. Tumor cells can also be eliminated by death receptor-dependent apoptosis. Activation of NK cells can induce death-inducing ligands, such as Fas ligand (FasL; also known as TNFSF6) and TNF-related apoptosis inducing ligand (TRAIL; encoded by TNFSF10) on the NK cell surface. When binding to their receptors on the target cells, these ligands further trigger activation of intracellular caspases in tumor cells to initiate cell death [45].

**Fig. 2:**
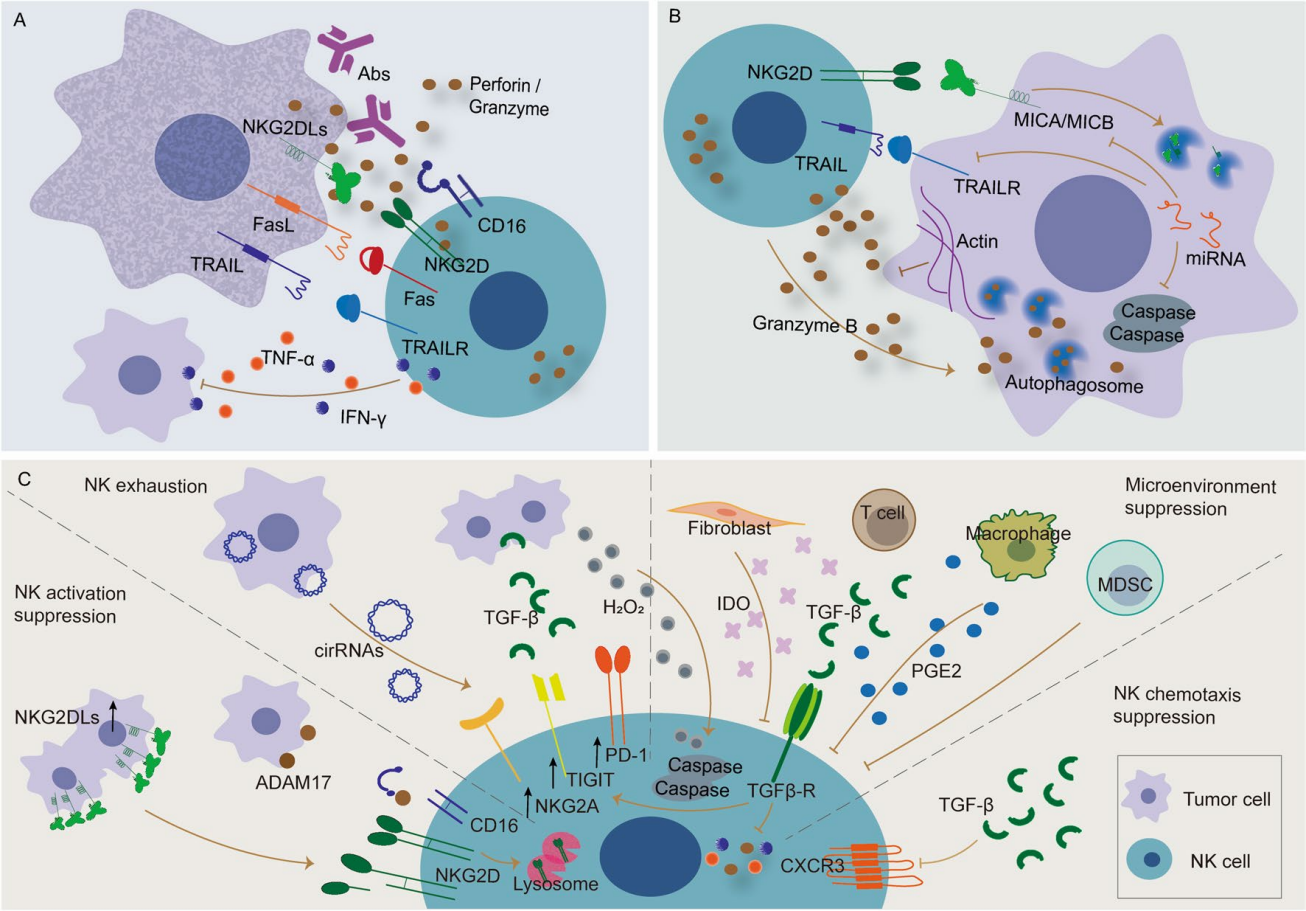
The interaction between NK cell and tumor cell. **A** Four mechanisms for NK cells to suppress tumors. NK cells can mediate cell death of the target tumor by CD16-dependent ADCC, releasing perforin and granzyme, secreting TNF-α and IFN-γ, or inducing apoptosis via Fas/FasL and TRAIL/TRAILR signals. **B** The resistance of tumor cells against NK cell cytotoxicity. Tumor cells initiate anti-apoptotic pathways, inactivate/eliminate toxicity granules, and shield corresponding activating ligands of NK cells. **C** Illustration of NK cell immunosuppression in the TME. The TME inhibits NK cell function via production of soluble modulators and hypoxic conditions to negatively regulate activation, mobility and effector function of NK cells, so promoting their apoptosis and exhaustion. Both **B** and **C** contribute to immune escape of tumor cells

Besides their direct dismantling, NK cells secrete multiple regulatory cytokines and chemokines that influence the activity of other innate and adaptive immune cells to demolish the target cells. Activated NK cells can secrete cytokine IFN-γ, TNF-α, IL-10, FLT3LG, and chemokine ligand CCL3, CCL4, CCL5, a process critical for antitumor immunity. Secretion of CCL5, XCL1 [46], and FLT3LG [47] may attract accumulation of conventional type 1 dendritic cells in tumors and improve patient overall survival. The secreted IFN-γ can promote the activation of macrophages and myeloid cells, initiate the differentiation of CD4^+^ T cells into Th1 cells, and induce MHC class II molecules on antigen-presenting cells [34]. IFN-γ also suppresses the expression of extracellular matrix protein fibronectin in tumors, reducing metastases [48]. Moreover, IFN-γ and TNF-α can directly induce cell cycle arrest and inhibit cell growth in tumors [49]. Release of TNF-α can subsequently promote B cell proliferation and stimulate differentiation of monocytes and macrophages [50, 51]. Notably, TNF-α can directly induce tumor cell necrosis [12]. The multiple aspects regarding how NK cells eliminate tumors are illustrated in Fig. 2A.

### Functional impairment and immunosuppression of NK cells

Despite their commanding ability to monitor and kill cancer cells, NK cells are susceptible to several factors that help cancer cells evade NK cell surveillance [52]. In general, the mechanisms of immune escape contain the intrinsic tumor heterogeneity, which disables cytotoxicity of NK cells or tames NK cells to be accomplices, and the complex immunosuppressive microenvironments, which quash the effectiveness of NK cells [53, 54]. Cancer cells might defend themselves against the attack of NK cells through activating anti-apoptotic pathways and inactivating/eliminating toxic granules released by NK cells [55–58]. For example, aggressive TP53-mutated breast cancers resisted apoptosis induced by TRAIL, FasL and granzyme B/perforin through overexpressing miR-519-3p to interfere the apoptotic signaling. Moreover, miR-519a-3p downregulated the NKG2D ligands ULBP2 and MICA on the surface of tumor cells, disqualifying their recognition by NK cells [55]. Proteolytic shedding of MICA/B is also a frequent immune evasion mechanism in various human cancers [56, 59]. Breast cancers were found to decrease susceptibility to NK cell lysis by degradation of NK-derived granzyme B in autophagosomes under hypoxia [57]. Another significant fraction of cancer cells responds to NK cell attack via a process termed ‘actin response’. The rapid and massive accumulation of F-actin near the immunologic synapse resulted in reduced intracellular levels of the cytotoxic protease granzyme B and lowered rates of apoptosis in tumor cells (Fig. 2B) [58].

Besides self-adjustment to avoid NK cell attack, cancer cells may release soluble immunomodulatory molecules, shed or engulf activating receptors on the surface of NK cells and increase inhibitory signals inside NK cells, thus mitigating their cytotoxicity. The outcomes of receptor endocytosis mediated by exposure to NKG2D ligand-expressing tumor cells have been manifested, showing eventual degradation of lysosomal receptors and impairment of NKG2D-mediated functions [60]. Another possible mechanism against NK cell attack is receptor shedding through proteolytic cleavage by tumor cells. For instance, CD16 cross-linked with antibodies undergone shedding from the cell membrane through action of disintegrin and metalloprotease-17, leading to decreased IFN-γ production [61, 62]. Immunomodulatory factors such as TGF-β, prostaglandin E2 (PGE2) and indoleamine 2,3-dioxygenase (IDO) are most reported, as they play immunosuppressive roles by reducing active receptors on NK cells and inhibiting NK development [21]. In a recent study, hepatocellular carcinoma (HCC) cells secreted exosomal circUHRF1 to suppress NK cell-derived IFN-γ and TNF-α levels by upregulating TIM-3 expression, presenting a novel mechanism for tumor resistance [63]. In patients with cancers, inhibitory molecules PD-1, CD96 and TIGIT are overexpressed on NK cells, responsible for NK cell exhaustion and reduced cytotoxicity. Blockades of these inhibitory signals with monoclonal antibodies (mAbs) have shown much improved tumor control [64]. The cytokine-inducible SH2-containing protein (CISH), a critical negative regulator of IL-15 signaling in NK cells, is proposed as an intracellular checkpoint. Nullifying CISH function in mice resulted in diminished metastasis of melanoma, prostate and breast cancers [65, 66].

Aside from intrinsic influences by tumor cells, the immunosuppressive tumor microenvironment (TME) constitutes another barrier against successful anti-tumor efficacy of NK cells [67]. Two important issues related to TME are the recruitment of effective NK cells and their persistence at the tumor core [68]. H_2_O_2_ produced within TME preferentially induced apoptosis of CD56^dim^ NK cells, contributing to limited cytolytic cell number in tumor site [69]. TGF-β was found to reduce chemokine receptor CX3CR1 expression on cytotoxic NK cells, decreasing NK cell infiltration [70]. Another issue is the impaired NK cell function due to the accumulation of suppressive factors in the TME. Different stromal cells present in TME, including cancer-associated fibroblasts, monocytes, myeloid-derived suppressor cells (MDSCs), tumor-associated macrophages (TAMs), and regulatory B cells, could negatively impact antitumor activity of NK cells [71, 72]. TGF-β, a key immunosuppressive molecule, might be produced by different types of immune cells in the TME. For instance, MDSCs produced TGF-β and H_2_O_2_ into tumors and suppressed NK cell function in head and neck squamous cell carcinoma [73]. Derived TGF-β from tumor-infiltrating monocytes or macrophages might impair the expression of IFN-γ, TNF-α, and Ki-67 in NK cells [74]. Also, TGF-β has been found to maintain the immunosuppressive effects on the cytolytic activity of NK cells through hyperactivating Smad3 while suppressing Smad7 and inhibiting the expression of NKG2D and MICA [38]. In addition, the tumor-caused nutritional competition is also known to affect antitumor immunity. NK cells required vitamin B6 (VB6) for intracellular glycogen breakdown, which serves as a critical energy source for NK cell activation. Pancreatic ductal adenocarcinoma cells suppressed NK cell toxicity by restricting VB6 accessibility [64]. Thus, understanding the possible mechanisms of immune evasion helps explore the evolving strategies regarding how to enhance antitumor effect of NK cells (Fig. 2C).

## NK cell-based therapeutic strategies in clinical settings

**Fig. 3:**
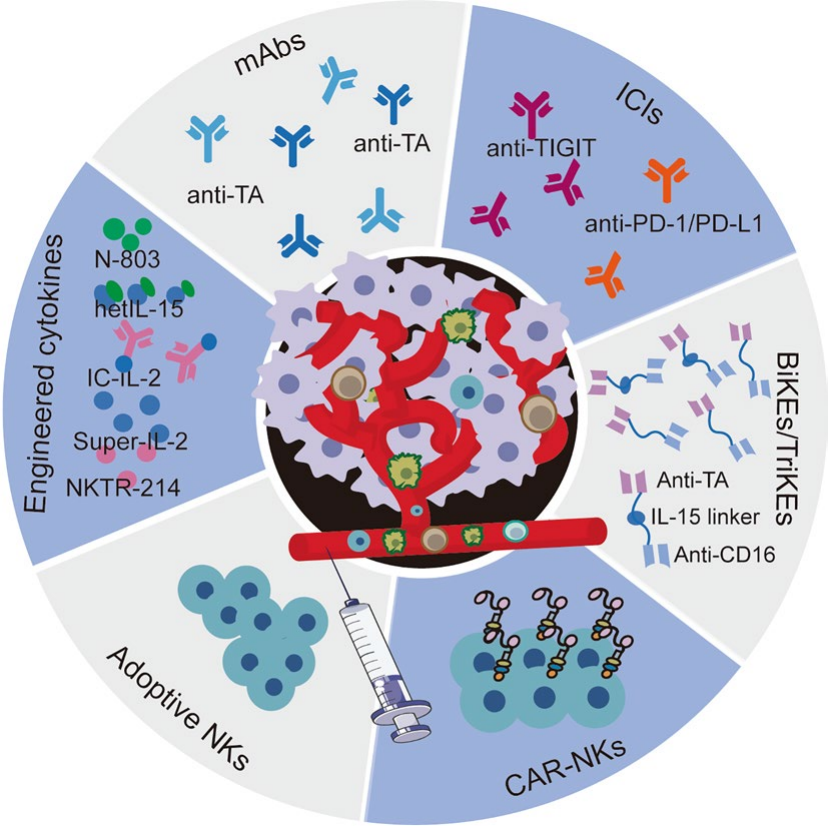
Therapeutic strategies of clinical NK cell therapies. Six strategies that mobilize NK cells to tumor treatment. Engineered cytokines, such as different active forms of IL-2 and IL-15, increase the potency compared with conventional cytokines; optimization of affinity between CD16 on NK cells and Fc fragment of mAb enhances the ADCC; checkpoint inhibitors (ICIs) such as PD-1 and TIGIT remove NK cells from the immunosuppressive environment, and promote their oncolytic function; specific killer engagers, BiKEs and TriKEs, make NK cells closer to their target cells and active them; autologous or allogeneic NK cells are safely used as adoptive cell therapy; CAR-NK cells enhance the efficiency of adoptive cell therapy. TA, tumor antigen

Strategies such as adoptive NK cell administration, cytokine supplement and mAb infusion have achieved encouraging results in recent clinical trials. Herein we elaborate these strategies by analyzing their advantages and shortcomings when applied in clinical settings (Fig. 3).

### Cytokine supplement

Cytokines as vital regulators strongly mobilize endogenous NK cells to kill tumors, which is economical and timely [75]. IL-2 is a growth factor for a wide range of leukocytes, including NK cells. Low-dose recombinant human IL-2 expanded peripheral Tregs in vivo, beneficial to avert graft-versus-host disease (GvHD) in the process of cell transplantation (NCT00529035) [76]. These doses of IL-2, however, were insufficient to stimulate endogenous cytotoxic cell populations to induce antitumor immune response (NCT00539695) [77]. High dose of IL-2 induced striking responses in patients with metastatic melanoma or renal cancer**,** but unacceptable adverse effects such as capillary leak syndrome (the most severe side effect of IL-2), constitutional symptoms and renal dysfunction were also observed [76].

Since systematic injection of high-dose IL-2 was accompanied by severe toxicities, engineered IL-2 was designed as a fusion protein to bind the Fc region of a humanized mAb and so to promote its chemotaxis. The immunocytokine (IC) IL-2, i.e., L19-IL2, in which IL-2 was fused to a humanized mAb L19, was capable of specific accumulation in tumor neovasculature, showing encouraging results in patients with metastatic melanoma [78]. The IC hu14.18-IL2, which bound to GD2 in neuroectodermal tumors, had antitumor effects involved in cytotoxic T cells and NK cells in murine models of melanoma and neuroblastoma. Clinical toxicities of hu14.18-IL2 therapy were generally transient and well controlled (NCT00590824) [79].

In addition, engineered IL-2 ‘superkine’ (also called super-2) with increasing binding affinity for IL-2 receptor subunits IL2Rβγ was used to drive increased proliferation and activation of CD8^+^ T and NK cells without unwanted expansion of Tregs in the TME [80]. The first-in-human study of NKTR-214, a novel IL2Rβγ-biased IL-2 pathway agonist, determined the recommended phase II dosage to be 6 μg/kg every three weeks [81]. Based on its biological activity and tolerability, NKTR-214 was being studied with an immune checkpoint inhibitor nivolumab, showing a therapeutic potential in solid tumors (melanoma, renal cell carcinoma, and non–small cell lung cancer) (NCT02983045) [82].

IL-15 and IL-15 variants were also administered to activate NK cells [83]. Patients received either intravenous (NCT01385423) or subcutaneous (NCT02395822) recombinant human IL-15 after lymphodepleting chemotherapy and haploidentical NK cell infusion, showing better rates in NK cell expansion and remission in vivo compared to those using IL-2, but it was associated with higher levels of IL-6 (a main cause for cytokine releasing storm or CRS) via subcutaneous rather than intravenous administration [84]. IL-15 is a membrane-bound heterodimer associated with IL-15 receptor alpha chains (IL15Rα) in such a way that it is trans-presented to cells expressing IL-2/IL-15Rβ and common γ chain receptor. Bioactive IL-15 is produced in vivo as a heterodimeric cytokine, comprising of IL-15 and IL-15Rα, termed heterodimeric IL-15 (hetIL-15) [85]. HetIL-15 administration increased NK cell tumoral infiltration in a mouse model [86]. N-803, previously known as ALT-803, is an IL-15 agonist mutant complexed to a dimeric IL-15Rα Sushi-Fc fusion protein. This fully humanized complex enhanced IL-15 biological activity and stability in vivo and promoted greater activation of NK cells than rIL-15 with less toxicity [87, 88]. Clinically, N-803 is being evaluated in combination with PD-1/PD-L1 inhibitors (NCT03228667). However, the lately clinical trial indicated that IL-15/N-803 promoted recipient CD8 T-cell activation, which in turn accelerated donor NK cell rejection. This means systemic IL-15 used to support allogeneic cell therapy may critically limit allogeneic cellular therapy (NCT03050216, NCT01898793) [89]. Thus, targeted delivery of IL-15 such as membrane-bound IL-15 constructed to CAR (e.g., CD19-iCasp9-IL15) could be another clinical choice (NCT03056339).

### Antibody and engager infusion

Antibody therapy is an off-the-shelf approach like cytokines to activate NK cells in vivo. Traditional approaches rely on antigen-specific binding mAb to activate NK cells via ADCC [90]. It was reported that anti-HER2 (human epidermal growth factor receptor 2) therapeutic antibody transtuzumab induced ADCC, mediated by HER2 on tumors and CD16/CD32 on NK cells, greatly improving the outcome of patients with HER2^+^ breast or gastroesophageal cancer [91]. However, patients expressing low-affinity isoforms of CD16 and CD32 demonstrated resistance to transtuzumab treatment. Margetuximab, which was Fc-modified to bind all allelic forms of CD16A and CD32A with increased affinity to induce ADCC, showed promising results in a first-in-human phase I study (NCT01148849) [92]. Other mAbs like nimotuzumab (NCT03554889) and rituximab (anti-CD20 mAb) (NCT03417414) partially mediated antitumor activity through ADCC mechanism.

To guide chemotaxis and promote adhesion of NK cells to the targets, engineered engagers were used to form an immunological synapse between NK and tumor cells, and to redirect autologous NK cells against tumors [93]. These engagers are small molecules, consisting of one fragment of single chain (scFv) specific for an NK cell receptor CD16 and one (bispecific killer engager, BiKE) or two (trispecific killer engager, TriKE) scFv with different specificities. CD16, the most potent activating receptor on NK cells, became the primary target of engagers [94]. In the preclinical studies, BiKE has been effectively used to target CD19/CD22 on B cell non-Hodgkin's lymphoma, CD33 on acute myeloid leukemia (AML) and myelodysplastic syndromes (MDS), EpCAM on various carcinomas, and CD133 on cancer stem cells [95].

Although BiKE has mediated some responses in various cancers, the limited proliferation and persistence of NK cells in the BiKE treatment need further improvement. A modified human IL-15 was incorporated to BiKE to form a 161533 (CD16/IL-15/CD33) TriKE, inducing expansion, priming, and survival of NK cells [96]. A phase I/II clinical trial of GTB-3550 (CD16/IL-15/CD33) TriKE is currently ongoing for the treatment of MDS, refractory/relapsed AML or advanced systemic mastocytosis. GTB-3550 TriKE is expected to induce NK cell function by targeting malignant cells as well as CD33^+^ MDSCs, which contributes to immunosuppression in tumors (NCT03214666).

Despite activating receptors on the target enhance NK cell-mediated tumor lysis, expression of ligands on tumors to inhibitory NK receptors, such as NKG2A, TIGIT and LAG3, can induce NK cell exhaustion and suppress their anti-tumor function. Thus, immune checkpoint associated mAbs are promising in reactivating exhausted NK cells in cancer immunotherapy [97–99]. Blocking the inhibitory receptor, such as NKG2A, enhanced tumor immunity by promoting NK functions in both mice and humans [100]. Monalizumab, a humanized anti-NKG2A antibody, is currently being tested in clinical trials as monotherapy or in combination with other antibodies (anti-HER2, or anti-PD-L1) [101]. A phase II clinical trial using monalizumab combined with cetuximab has shown encouraging efficacy in patients with squamous cell carcinoma of head and neck (NCT26435509).

### Adoptive NK cell administration

NK cells destroy tumors in an MHC-independent manner without prior sensitization or immunization [102]. Therefore, NK cell transfer is considered as a practical option to enhance autologous immunity. In the past years, autologous NK cell transfer has been experimentally performed to treat certain types of cancers. A phase I clinical trial has demonstrated the safety and efficacy of multiple infusions of activated and expanded NK cells in combination with anti-myeloma drugs in myeloma patients (NCT02481934). Studies showed that autologous NK cells could be more effective if tumor cells lack HLA ligand to KIR expressed by the transferred NK cells [103]. Despite successful NK cell engraftment in certain types of cancers, results with unmodified autologous NK cells remained discouraging [104, 105]. This could be due to the anticancer treatments received by the patients before NK collection, which indirectly dampened the function and status of autologous NK cells. Additionally, self-HLA signals in tumor cells were likely to inhibit the antitumor activity of autologous NK cells as referred before [106]. Allogeneic NK cells or CAR-NK cells, however, may overcome these limitations. Clinical evidences showed that allogenic NK cells were more efficacious than autologous NK cells by taking advantage of the ‘missing-self’ recognition [107].

**Fig. 4:**
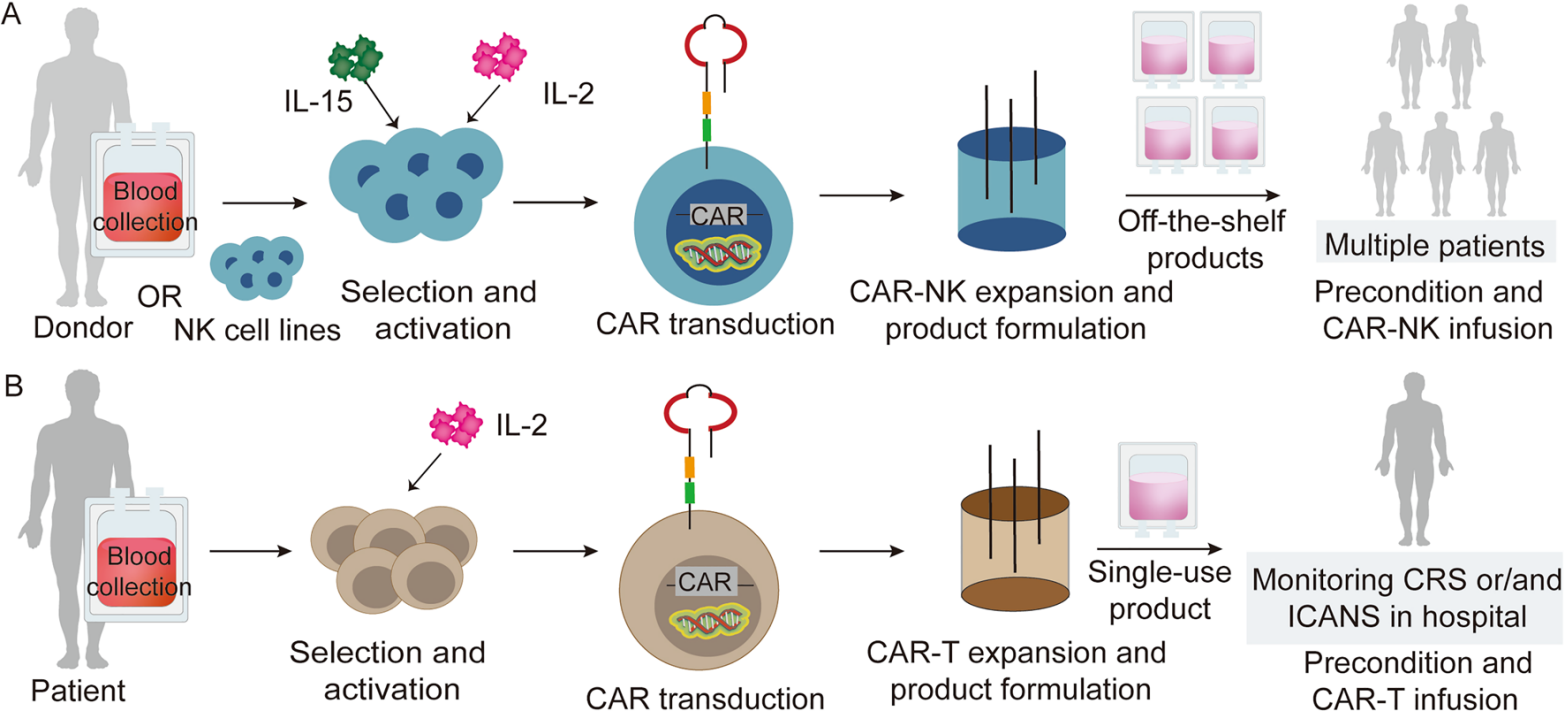
CAR-NK and CAR-T cell therapies. **A** NK cells are isolated from the blood of a patient or a donor, cultured and activated under GMP conditions. The primary NK cells or NK cell lines (especially NK-92 cell) are genetically engineered to express the CAR construct by retrovirus or lentivirus. After ex vivo expansion of CAR-NK cells, the final products are formed and cryopreserved as the ‘of-the-shelf’ products. After KIR-HLA matching test with donor NK cells, patients receive CAR-NK product treatment. **B** T cells are isolated from blood of the patient, activated, and then transduced with CAR expression. After ex vivo expansion of CAR-T cells, they are formulated into the single-use product. The patient undergoes a conditional chemotherapy, and the CAR-T cell product is directly infused. The patient received CAR-T product needs to be hospitalized to monitor CRS or ICANS for several weeks. CRS, cytokine releasing storm; ICANS, immune effector cell-associated neurotoxicity syndrome

CARs play an important role in enhancing antitumor activity of NK cells [108]. CARs can be constructed by linking variable scFv of mAbs against surface molecules expressed on tumor cells to a transmembrane domain by a hinge. The intracellular signaling chains (e.g., CD3ζ) are often linked to costimulatory domains encoding CD28 or 4-1BB (CD137) to fully activate NK cells (Fig. 4) [109]. In a Raji B-cell lymphoma model of NOD-SCID IL2Rγ^null^ (NSG) mice, NK-92 cells were engineered by lentivirus to express CARs targeting CD19, inhibiting cancer progression [110]. Current phase I/II clinical trials that engineer NK-92 cell lines with CARs to target CD19 (NCT02892695), CD33 (NCT02944162), CD7 (NCT02742727) on acute myeloid leukemia (AML) and acute lymphocytic leukemia (ALL) cells are ongoing. To ensure the safe usage of highly active NK-92 cells, large dosage of radiation is applied to treat CAR-NK-92 cells before clinical application, which prevents their excessive expansion but also reduces their antitumor activity and IFN-γ production [111]. Instead, primary NK cells like cord blood-derived anti-CD19 CAR-NK cells without irradiation demonstrated a satisfactory efficacy in vivo without major side effects [112].

Cell line originated CAR-NK cells could be risky for their excessive expansion and long-time retention in vivo, but non-cell line originated CAR-NK cells were not persistent in vivo [16]*.* Thus, approach to extend persistency of primary NK cell in vivo is necessitated. Based on the preclinical studies, IL-15 improved persistency of NK cells in vivo, and clinical trial of CAR-NK cells expressing IL-15 was under evaluation (NCT03056339) [113]. Furthermore, other approaches, such as MLNK, demonstrated long-term persistence for weeks to months in vivo after pre-activation [114]. In a first-in-human phase I clinical trial, adoptively transferred MLNK proliferated and expanded in AML patients and responded robustly against leukemia (NCT01898793). Half of evaluable AML patients (4 out of 8) achieved complete remission at day 28 after MLNK adoptive transfer (NCT03068819) [115]. In addition, MLNK extracted from the peripheral blood were modified to express an anti-CD19 CAR (CD19-CAR-ML), which displayed significantly increased IFN-γ production, degranulation, and improved specific responses against their autologous lymphomas compared with non-specific control CAR-MLNK or conventional non-ML CAR-NK cells [116].

Furthermore, pluripotent stem cell-derived CAR-NK cells are currently undergoing clinical development [117]. Briefly, induced pluripotent stem cells (iPSCs) are engineered with CARs, and then are differentiated into CD34^+^ CAR-hematopoietic progenitor cells (CAR-HPCs). Subsequently, the CAR-HPCs were differentiated into iPSC-derived CAR-NK (i.e., CAR-iNK) cells using NK cell initiating cytokines (IL-3, IL-7, IL-15, SCF, FLT3L). For further expansion, CAR-iNK cells were co-cultured with irradiated and engineered K562 cells [118]. Besides, many other functional genes can be engineered to iPSCs to further improve the functions of CAR-iNK cells [119]. For example, quadruple gene-engineered CAR-iNK cells were designed for dual targeting against multiple myeloma (MM) through the introduction of specific CAR for B cell maturation antigen (BCMA) and high-affinity non-cleavable CD16 to augment ADCC activity. Additionally, these cells expressed a membrane-bound IL-15 fusion molecule to enhance persistence, along with knockout of CD38 to prevent antibody-mediated fratricide and enhance NK cell metabolic fitness [120]. In the preclinical models, these quadruple gene-engineered CAR-iNK cells consistently demonstrated durable antitumor activity independent of exogenous cytokine support. More importantly, when combined with anti-CD38 mAb, these cells achieved augmented ADCC activity and anti-tumor effects towards MM [120]. These results supported the development of a phase I clinical trial of these CAR-iNK cells as monotherapy or in combination with daratumumab (anti-CD38 mAb) for treatment of patients with refractory/relapsed MM (NCT05182073).

## Preclinical and clinical administration of adoptive NK cells in solid tumors

Solid tumors are always challenging to treat, based on patient experience from radiotherapy, chemotherapy, to immunotherapy or even CAR-T therapy. Recent data from preclinical experiments and clinical trials of NK cell-based cancer therapy have proven adoptive NK cell transfer to be a promising therapy in solid tumors.

### Non-genetically modified NK cell infusion

Non-genetically modified NK cell infusion has been used in many types of solid tumors, including neuroblastoma, renal, ovarian, pancreatic, breast, and lung cancers (Table 1). Different therapeutic effects have been observed, and here we elaborate the safety and efficiency of NK cell therapy in solid tumors as follows.

**Table 1:** Clinical outcomes of non-genetically modified NK cell therapy in solid tumors

Condition	Source	Ex vivo preparation	Combined treatment	# of patients	Response	Initiated date	Status	Phase	Identifiers
Advanced NSCLCs	Allogeneic/PB	A human high-activity natural killer cell in vitro preparation kit	Pembrolizumab	109	Patients had longer survival than patients treated with chemotherapy alone	07-01-2016	Completed	I/II	NCT02843204 [128]
Advanced Solid tumors	Allogeneic/PB	irradiated autologous feeder cell activated	Chemotherapy	17	SD in 7 patients; PD in 8 patients PFS in patients with SD was 4 months	09-2010	Completed	I	NCT01212341 [126]
Advanced Solid tumors	NK92 cell	IL-2	Chemotherapy (1 with HSCT)	15	SD in 1 patient; MR in 2 patients; PD in 12 patients;	–	Completed	I	[207]
Childhood metastatic Solid Tumor	Allogenic/PB	IL-15 stimulated	HSCT	3	CR in 1 patient; 2 patients died after HSCT	01-2011	Terminated	I/II	NCT01337544
Digestive cancer	Autologous/PB	OK432, IL-2/FN-CH296 induced T cells	–	14	NR in all patients	–	Completed	I	[10]
Neuroblastoma	Haploidentical	IL-2 preactivated	anti-GD2 mAb/ cyclophosphamide	35	CR in 5patients; PR in 5 patients; NR in 17patients; PD in 8 patients	04-02-2009	Completed	I	NCT00877110 [208]
Neuroblastoma	Haploidentical	Isolated	Chemotherapy/ anti-GD2 mAb/GM-CSF/IL-2	13	CR in 4patients; PR in 4 patients; SD in 5 patients	–	Completed	–	[127]
Fallopian tube cancer; Ovarian cancer; Peritoneal cavity cancer	Allogeneic	–	IL-2	14	PR in 3 patients; SD in 8 patients; PD in 1 patient	03-2008	Terminated	II	NCT00652899
Ovarian cancer; Breast cancer	Allogeneic/ PB	CD3 depleted apheresis	IL-2(7 with TBI)	20	PR in 4 patients (all ovarian); SD in 12 patients (8 ovarian and 4 breast); PD in 3 patients (1 ovarian and 2 breast)	–	Completed	II	[209]
Advanced metastatic breast cancer	Allogenic	Isolated	Chemotherapy/IL-2(s.v.)	6	1 patient died within 100 days; 5 patients died after 100 days;	04-2006	Terminated	II	NCT00376805
Solid tumors	Allogenic/PB	K562-mb15-41BBL activated	HSCT	9	CR in 2 patients; 4 patients remain alive	01-29-2011	Completed	I	NCT01287104 [123]
Solid tumors	Autologous/PB	IL-2 preactivated	Chemotherapy	10	SD in 3 patients; PR in 1 patient; PD in 6 patients	–	Completed	I/IIa	[121]
Melanoma; Renal cell carcinoma	Haploidentical	CD3 depleted apheresis/IL-2 preactivated	–	57	CR in 5 patients (all AML)	–	Completed	–	[125]
Metastatic melanoma Metastatic kidney cancer	Autologous/PB	Isolated	Chemotherapy/IL-2	8	CR in 0 patients; PR in 0 patient	05-2006	Completed	–	NCT00328861
Solid tumors	Haploidentical/ PBSC	T cell depleted	Chemotherapy	9	Engraftment failure in 3 patients	08-2008	Terminated	I/II	NCT00582816
Ovarian cancer; Fallopian tube cancer; Primary peritoneal cancer; Breast cancer	Allogenic	–	Cyclosporine/Methylprednisolone/ IL-2	13	PR in 8 patients	07-2010	Completed	–	NCT01105650

CR: completed remission; NR: no response; MR: mixed response; mAb: monoclonal antibody; OS: overall survival; PFS: progress free survival; PD: progressive disease; PR: partial response; PBSC: peripheral blood stem cell; SD: stable disease

Regarding safety, autologous or allogeneic transplantation might yield different outcomes. In a clinical trial using autologous transplantation, NK cells from CD3-depleted  peripheral blood mononuclear cells (PBMCs) were infused back to patients with solid tumors. As a result, no severe toxicities nor adverse outcomes were found [121]. However, efficiency of this autologous NK cell therapy was very limited. Only 3 of 10 patients achieved stable condition and none had complete remission [121]. In patients with digestive tract cancer, autologous administrations of NK cells were safe and well tolerated, but no clinical responses were obtained in these patients [10]. Whether the activated state due to ex vivo preparation [10, 121] or self-signal suppression between tumor cells and NK cells [122] resulted in the low response rate remained unidentified. Nevertheless, given safety of autologous NK cell infusion, an attempt at improving their antitumor effect by combining with other treatment was hopeful [10]. Comparatively, although allogeneic NK cells were considered not causing GvHD [122], they might augment underlying T-cell alloreactivity. As a result, 5 of 9 transplant recipients still experienced acute GvHD following allogeneic NK cell infusion, and Grade 4 GvHD was observed in 3 patients. GvHD was more common in matched unrelated donor and matched sibling donor recipients with higher donor CD3 chimerism [123]. Nevertheless, successful cases have been reported in malignant hematologic cancers after haploidentical NK cell transplantation when HLA ligands against the inhibitory KIRs present in the donor were absent in the recipient (KIR-HLA receptor-ligand mismatch) [124].

Regarding efficiency, productive expansion of adoptively transferred NK cells in vivo might contribute to cancer remission in patients. Expansion was dependent on the level of lymphocytes and the potent immunosuppression in recipients induced by cyclophosphamide and fludarabine. Patients with infused NK cells after high dosage of cyclophosphamide and fludarabine showed an obvious rise in endogenous IL-15, signifying expansion of donor NK cells. Five of 19 poorly prognostic patients with AML achieved complete hematologic remission, although no progress was reported in solid tumors in this trial [125]. In addition, persistency of administrated NK cells may be affected by allo-specific immune responses in the host [126]. Antibodies specific for donor NK cells after repeated injections appeared more than those after a single injection in patients. Allogenic NK cells persisted for up to 4 days after a single injection and for several hours to 3 days after repeated injections. Patients who received repeated injections of higher doses of NK cells seemed to have better outcomes than patients injected one time with a lower dose, although this difference was not statistically significant [126]. To further improve NK cell efficacy, non-genetically modified NK cells are combined with mAbs in clinical trials treating solid tumors. NK cells can be safely combined with anti-GD2 mAb that binds to disialoganglioside (GD2) expressed on neuroblasts, for an enhanced treatment of recurrent or refractory neuroblastoma. In 11 of 13 patients who received both NK cell and anti-GD2 mAb, the response rate was 61.5% and 5 had stable conditions [127].The anti-PD-1 antibody pembrolizumab combined with NK cells yielded an improved survival benefit in patients with advanced PD-L1^+^ NSCLC [128]. Patients who received combination therapy had longer survival than did patients who received pembrolizumab alone. Moreover, the patients treated with multiple courses of NK cell infusion had better overall survival (18.5 months) than did those with a single course (13.5 months) [128]. It was also observed that PD-1 expression was lower in patients who received combination therapy, implying a better recovery of activated NK cells [128]. These results suggested that adoptive NK cells together with mAbs might synergistically enhance antitumor effects and improve patient benefits.

### Preclinical experiments and clinical trials using CAR-NK cells

CAR-NK cells have been tested safe and efficient in relapsed or refractory CD19 positive lymphoid tumors (NCT03056339/NCT03056339). Numerous clinical CAR-NK cell trials dedicated to hematological TAs are in full swing (Table 2). Although clinical trials of CAR-NK cell in solid tumors yielded no results, preclinical experiments have achieved satisfied outcomes [129].

**Table 2:** Clinical trials of CAR-NK cell therapy in hematological and solid tumors

Type	Target	Condition	NK source	CAR structure	Phase	Initiated date	Status	Location	Identifiers
Hematological malignancies	CD19	BCL	CB-NK	CD28ζ + iCasp9 + IL15	I/II	06-21-2017	Completed	USA	NCT03056339
	CD19	r/r acute ALL	Unknown	Unknown	I	07-21-2002	Completed	China	NCT05563545
	CD19	Leukemia/Lymphoma	NK92	TCRζ + CD28 + 4-1BB	I/II	09-2016	Unknown	China	NCT02892695
	CD19	B-cell NHL	Haploidentical NK	Unknown	I	05-01-2021	Recruiting	China	NCT04887012
	CD19	BCL	Allogeneic NK	Unknown	I	08-01-2022	Recruiting	USA	NCT05020678
	CD19	ALL/CLL/NHL	CB-NK	Unknown	I	04-10-2021	Recruiting	China	NCT04796675
	CD19	BCL	CB-NK	CD28ζ + iCasp9 + IL15	I/II	10-03-2019	Withdrawn	USA	NCT03579927
	CD19	B-cell NHL	Unknown	Unknown	I	12-17-2020	Not yet recruiting	China	NCT04639739
	CD19	BCL	Unknown	Unknown	I	03-2019	Not yet recruiting	Unknown	NCT03690310
	CD19	ALL/CLL/ BCL	Unknown	Unknown	I	03-10-2021	Recruiting	China	NCT04796688
	CD19	r/r diffuse large B cell lymphoma	Unknown	Unknown	I	03-01-2023	Recruiting	CD19	NCT05673447
	CD19	r/r BCL	Unknown	Unknown	I	05-25-2022	Recruiting	China	NCT05410041
	CD19	r/r B cell hematologic malignancies	Unknown	Unknown	I	12-01-2022	Recruiting	China	NCT05645601
	CD19	r/r BCL/ r/r CLL	Allogeneic NK	Unknown	I	03-01-2023	Recruiting	China	NCT05739227
	CD19	BCL	Unknown	Unknown	I/II	10-01-2022	Withdrawn	China	NCT05570188
	CD19	B-cell NHL	CB-NK	Unknown	I	09-10-2022	Recruiting	China	NCT05472558
	CD19	r/r B cell malignancies	Unknown	Unknown	I	04-10-2021	Unknown	Unknown	NCT04796675
	CD19	B-CL	Unknown	Unknown	I/II	12-08-2022	Recruiting	China	NCT05654038
	CD19	BCL	Unknown	CD28ζ-iCasp9-IL15-	I/II	10-03-2019	Withdrawn	USA	NCT03579927
	CD19	r/r BCL/ r/r CLL	iPSC	CAR-hnCD16-IL15	I	03-19-2020	Terminated	USA	NCT04245722
	CD19	NHL	iPSC	CAR-hnCD16-IL15	I	09-22-2020	Completed	USA	NCT04555811
	CD19	B-cell malignancies	iPSC	CAR-sIL-15	I	01-24-2023	Recruiting	USA	NCT05336409
	CD19/ CD70	B-cell NHL	CB-NK	Unknown	I	12-15-2022	Recruiting	China	NCT05667155
	CD19/ CD70	r/r B-cell NHL	CB-NK	Unknown	I/II	01-18-2023	Recruiting	China	NCT05842707
	CD19/ CD22	BCL	Unknown	Unknown	I/	02-01-2019	Unknown	Unknown	NCT03824964
	CD22	Refractory BCL	Unknown	Unknown	I	03-2019	Unknown	Unknown	NCT03692767
	CD33	AML	NK92	TCRζ + CD28 + 4-1BB	I/II	10-2016	Unknown	China	NCT02944162
	CD33	AML	Unknown	Unknown	I	12-23-2021	Not yet recruiting	China	NCT05008575
	CD33	r/r AML	NK92	Cd28 + CD137 + CD3ζ	I/II	10-2016	Unknown	China	NCT02944162
	CD33	r/r AML	Unknown	Unknown	I	12-23-2021	Unknown	Unknown	NCT05008575
	CD123	r/r AML	Allogeneic NK	Unknown	I	10-01-2022	Recruiting	China	NCT05574608
	CD123	r/r AML or BPDCN	Unknown	Unknown	I/II	08-31-2023	Recruiting	China	NCT06006403
	CD123	r/r AML	Unknown	Unknown	I	12-30-2023	Recruiting	China	NCT06201247
	BCMA	MM	NK92	Unknown	I/II	05-2019	Recruiting	China	NCT03940833
	BCMA	MM	CB-NK	Unknown	I	10-01-2021	Not yet recruiting	China	NCT05008536
	BCMA	r/r MM/ Plasma cell leukemia	Unknown	Unknown	I	07-04-2023	Recruiting	China	NCT06045091
	BCMA	r/r MM	Unknown	Unknown	I	11-13-2022	Recruiting	China	NCT05652530
	BCMA	r/r MM	Unknown	Unknown	I	04-30-2024	Not yet recruiting	Iran	NCT06242249
	BCMA	r/r MM	Unknown	Unknown	I	11-10-2021	Recruiting	USA	NCT05182073
	BCMA	r/r MM	iPSC	CAR-hnCD16-IL-15	I	11-24-2021	Not yet recruiting	USA	NCT05182073
	CD5	r/r Hematological malignances	CB-NK	Including IL-15	I/II	04-22-2024	Not yet recruiting	USA	NCT05110742
	CD7	Leukemia/Lymphoma	NK92	TCRζ + CD28 + 4-1BB	I/II	03-2016	Unknown	China	NCT02742727
	NKG2D	AML/MDS	Allogeneic NK	Unknown	I	09-21-2020	Recruiting	USA	NCT04623944
	NKG2D	Relapsed /refractory AML	CB-NK	Unknown	Unknown	10-13-2021	Terminated	China	NCT05247957
	NKG2D	Relapsed /refractory AML	Unknown	Unknown	Unknown	03-2023	Recruiting	China	NCT05734898
	CLL1	r/r AML	Unknown	Unknown	I	03-15-2024	Recruiting	China	NCT06307054
	CLL1	r/r AML	iPSC NK	Unknown	I	09-10-2023	Recruiting	China	NCT06027853
	CD33/CLL1	Relapsed/refractory AML or MRD AML	iPSC NK	Unknown	Unknown	08-10-2023	Not yet recruiting	China	NCT05987696
	CD33/ CLL1	AML	Unknown	Unknown	I	11-30-2020	Unknown	China	NCT05215015
Solid tumors	NKG2D	Solid tumor	Autologous/allogeneic NK	Unknown	I	01-02-2018	Unknown	China	NCT03415100
	NKG2D	Refractory metastatic colorectal cancer	Unknown	Unknown	I	12-10-2021	Recruiting	China	NCT05213195
	NKG2D	Platinum-resistant recurrent ovarian cancer	Unknown	Unknown	I	03-2023	Recruiting	China	NCT05776355
	NKG2D	Metastatic solid tumors	Unknown	Unknown	I	01-02-2018	Unknown	China	NCT03415100
	5T4	Advanced solid tumors	Unknown	Unknown	I	12-30-2021	Unknown	China	NCT05194709
	ROBO1	Solid tumor	Unknown	Unknown	I/II	05-2019	Recruiting	China	NCT03940820
	ROBO1	Pancreatic cancer	Unknown	CD3ζ + 41BB + iCas9	I/II	05-2019	Unknown	China	NCT03941457
	ROBO1	Malignant tumor	Unknown	Unknown	I/II	05-2019	Recruiting	China	NCT03931720
	PSMA	Prostate cancer	Unknown	Unknown	I	03-2019	Not yet recruiting	Unknown	NCT03692663
	MSLN	Ovarian cancer	PB-NK	Unknown	I	03-2019	Not yet recruiting	Unknown	NCT03692637
	MICA/MICBα3	Advanced solid tumors	iPSC	CAR-hnCD16-IL15	I	05-31-2022	Terminated	USA	NCT05395052
	PD-L1	GEJ cancer/ HNSCC	Unknown	Unknown	II	12-14-2021	Recruiting	USA	NCT04847466
	HER 2	Glioblastoma	NK92	CAR5.28.z	I	12-01-2017	Recruiting	Germany	NCT03383978
	MUC1	Solid tumor	Unknown	Unknown	I/II	07-2016	Unknown	China	NCT02839954
	DLL3	Relapsed and refractory extensive small cell lung cancer	Unknown	Unknown	I	09-01-2022	Recruiting	China	NCT05507593
	CLDN6/GPC3/MSLN /AXL	Advanced solid tumors	Unknown	Unknown	I	06-01-2022	Recruiting	China	NCT05410717
	TROP2	Solid tumors	CB-NK	Including IL-15	I	10-24-2023	Recruiting	USA	NCT06066424
	TROP2	MRD CRC	CB-NK	Including IL-15	I	–	Not yet recruiting	USA	NCT06358430
	CD70	Advanced renal cell carcinoma, mesothelioma, or osteosarcoma	CB-NK	Including IL-15	I/II	03-29-2023	Recruiting	USA	NCT05703854
	Unknown	Ovarian epithelial carcinoma	Unknown	Unknown	I	08-13-2024	Not yet recruiting	China	NCT05856643
	Unknown	Advanced triple negative breast cancer	Unknown	Unknown	I	02-01-2023	Not yet recruiting	China	NCT05686720

BPDCN: blastocytic plasmacytoid dendritic cell neoplasm; BCL: B-cell Lymphoma; CLL: chronic lymphocytic leukemia; CB: cord blood; CRC: colorectal cancer; GEJ: gastroesophageal junction cancers; HNSCC: head and neck cancer; NHL, non-Hodgkin lymphoma; r/r: refractory or relapsed; MRD: minimal residual disease

Pancreatic cancer, especially pancreatic ductal adenocarcinoma (PDAC), is one of the highly malignant cancers with early local invasion and distant metastasis [130]. In a case report from clinical trial (NCT03941457), the patient with primary PDAC and liver metastasis received Robo1-directed 4-1BB.CD3ζ.iCas9.CAR-NK92 injection. Pancreatic lesion and liver metastasis were controlled within 5 months, although the patient had moderate fever after intravenous injection. The overall survival time of the patient was 8 months [131]. In addition, Robo1-CAR-NK92 cells exhibited potent and targeting cytotoxicity against glioma and neuroblastoma cells. Robo1-directed 4-1BB.CD3ζ.CAR-NK92 cells were constructed by lentivirus transfection and tested 98.89% positive after sorting and expansion. The specific lysis of Robo1-CAR-NK92 cells against both glioma U87-MG cells and neuroblastoma SH-SY5Y cells was close to 100% at NK cells to target ratio of 1:1 in vitro [132]*.* These encouraging data motivated other two phase I/II clinical trials with aim to assess the safety and efficacy of ROBO-1-directed CAR-NK cell therapy in PDAC and other solid tumors expressing ROBO-1, which are currently recruiting patients in China (NCT03940820/NCT03931720).

HER2 is extensively expressed on patients with solid tumors [133]. A stable clonal NK-92 cell line expressing a humanized CAR based on HER2-specific antibody FRP5 with CD28 and CD3ζ signaling domain (CAR5.28.z) was generated. A good manufacturing practice (GMP)-compliant procedure to expand therapeutic doses of NK-92/5.28.z cells was successfully established [134]. While these NK-92/5.28.z cells efficiently lysed HER2-expressing tumor cells in vitro, their specific recognition and cytotoxicity of HER2-positive tumor cells were obtained in vivo, resulting in selective enrichment of NK-92/5.28.z cells in orthotopic breast carcinoma xenografts, and reduction of pulmonary metastasis in a renal cell carcinoma model, respectively [135]. These NK-92/5.28.z cells also showed potentials to improve immunotherapy of high-risk HER2-positive rhabdomyosarcoma (RMS) in 2D and 3D culture models [136]. Despite favorable primary data, no clinical trial has yet been approved to apply CAR-NK therapy in patients with breast cancer and high-risk RMS. Only a phase I clinical trial of NK-92/5.28.z cells (NCT03383978) is recruiting glioblastoma multiforme (GBM) patients in Germany. In preclinical GBM experiment, the median survival of symptom-free survival was remarkably extended from 73 days to 200.5 days compared with parental unmodified NK cells upon repeated injection of NK-92/5.28.z cells in orthotopic GBM xenograft models of immunodeficient NSG mice. In immunocompetent mice, local therapy with NK-92/5.28.z cells resulted in cures of transplanted syngeneic GBM in 4 of 5 mice carrying subcutaneous tumors and 5 of 8 mice carrying intracranial tumors [137].

Delta-like ligand 3 (DLL3) has been reported to be overexpressed in small cell lung cancer (SCLC) and may be a rational target for CAR-NK immunotherapy. DLL3-CAR-NK-92 cells induced tumor regression in a subcutaneous tumor model of SCLC and an H446-derived pulmonary metastasis tumor model [138]. Mesothelin (MSLN) is a well-known tumor associated antigen overexpressed in many solid tumors and may be an ideal immunotherapy target [139]. MSLN-CAR-NK cells can effectively eliminate gastric cancer cells in both subcutaneous and intraperitoneal tumor models. They could also significantly prolong the survival of intraperitoneally tumor-bearing mice. More importantly, the potent antitumor effect and considerable NK cell infiltration were observed in the patient-derived xenograft treated with MSLN-CAR-NK cells [140]. In addition, MSLN-CAR NK92 cells effectively eliminated ovarian cancer cells in both subcutaneous and intraperitoneal tumor models and significantly prolonged the survival of intraperitoneally tumor-bearing mice [141]. A phase I clinical trial of CAR-NK cells targeting MSLN for ovarian cancer treatment is undergoing (NCT03692637). While the results of the first CAR-NK cell clinical trials in solid tumors still await, accumulating preclinical data have indicated that CAR-NK therapy owns great potential in treatment of solid tumors [129].

### NK cells derived extracellular vesicles as an alternative to cell therapy

Although NK cell infusion has achieved initial success in patients with solid tumors, obstacles such as infrequent infiltration to tumor core and TME still debilitate its clinical applications [142]. Extracellular vesicles (EVs), as a cell-free product, can be considered as an alternative or auxiliary agent of NK cell therapy, with a hope to overcome the indomitable barriers in NK cell therapy, especially for solid tumor treatment [143].

Extracellular vesicles are plasma membrane-bound vesicles, containing a variety of bioactive molecules. They are intercellular messengers which can be released by donor cells and received by recipient cells [144]. NK cell-derived EVs have been isolated from culture supernatant of NK-92 cells, PBMCs or primary NK cells [145]. Human NK cells can release EVs in both unstimulated and stimulated condition [146]. NK cell-derived EVs express typical NK biomarkers CD56, NK activating receptor NKG2D, natural cytotoxicity receptors NKp30, NKp46 and NKp44, as well as killer proteins FasL and perforin, etc. [147]. Evidence showed that receptor-ligand mediated interaction (e.g., Fas/FasL) led to apoptosis of tumor cells in a caspase-dependent manner [148]. The killer protein perforin and granzyme induced apoptosis upon fusion of NK cell derived EVs with target cells in both caspase-dependent and caspase-independent manners [149]. NK cell derived EVs are exclusively functional against activated immune cells, suggesting that NK EVs have both antitumor and immune homeostatic activities [146, 150]. Moreover, NK EVs as a nanosized delivery vehicle carrying chemotherapy drugs or therapeutic nucleic acids were reported to enhance their antitumor effect in hematologic malignancies and solid tumors [151, 152]. EVs derived from a variety of NK cells upon different stimuli may present different activities, showing a controllable method to harness their functions [153, 154].

## Comparison of NK and T cell based-cancer therapies

For the time being, adoptive cellular immunotherapies mainly include CAR-T, tumor infiltrating lymphocytes, T-cell receptor-engineered T cells, and engineered NK or dendritic cells [155]. Given an intensive study of T-cell therapy in the recent years, especially CAR-T cell therapy, we next compare the operational procedures between T and NK cell-based cancer therapies, and analyze their advantages and disadvantages, followed by their respective challenges and possible solutions.

### Clinical procedures of adoptive T and NK cell-based cancer therapies

Currently, clinical trials of adoptive cell therapies are only permitted for patients who lack responses to existing treatments or have no other treatment options [156]. Before clinical trials, patients who are about to be transplanted with autologous immune cells receive risk evaluation. It may fail to collect cells with sufficient quality or/and quantity from patients, or to tolerate the following lymphodepletion before cell re-infusion. For allogeneic immune cell transplant, recipient and donor will receive human histocompatibility antigen matching assessment [107]. In NK cell transfer, additional KIR and HLA-I antigen mismatching assessment is required. As KIR and HLA-I genes are key players to decide the efficacy of allogenic NK cell transfer, gene mismatching may directly affect clinical response [157]. For example, progression-free survival (PFS) was assessed in patients with hematological malignancies after hematopoietic stem cell transplantation (HSCT) from unrelated donors. Increased PFS was shown in patients with missing KIR-HLA pair [158]. After the evaluation, apheresis products are collected and purified, CAR genes are delivered by retrovirus or lentivirus transduction, and specific products which express CARs are then sorted, activated and expanded in vitro [159]*.* In general, the production of CAR products takes 2–4 weeks [160].

Before administration of CAR products, patients are hospitalized at least 1 week ahead of lymphodepletion. Chemotherapy (e.g., fludarabine phosphate and cyclophosphamide) and total body irradiation, help quench the immune system of patients from resisting the donor cells [161]. Although resistance is not an issue for autologous cells, lymphodepletion can extend the persistence of re-infused cells and diminish immunosuppressive MDSCs and regulatory T cells (Treg cells), generating a favorable environment for adoptive cell functioning [125]. At this moment, patients need second evaluation, because they may be no longer qualified at the scheduled time of administration. For example, a patient’s condition may be worsened during product preparation, resulting in intolerance to the trial procedure or an expected survival time shorter than the study duration. After cell infusion, the evaluation of objective response and disease progression are next needed under consensus guidelines, such as Response Evaluation Criteria in Solid Tumors [162]. Meanwhile, CAR related cells are “living drugs” for which long-term efficacy and follow-up safety assessments are required to evaluate the survival and persistence of immune cells in vivo [163]*.* During this period, technologies enabling physicians to immediately control the infused cells in case of adverse events are required. The engineered CAR constructs to express suicide genes like the inducible caspase 9 (iCasp9) allowed the selective depletion of administrated cells once toxicity occurred [164].

Differences exist in the clinical procedure between CAR-T and CAR-NK cell therapies. For autologous CAR-T therapy, collection of autologous T cells for transduction can be insufficient due to prior chemotherapy in patients. Allogeneic CAR-T cells might overcome this problem and obviate the production delay in patients with progressive disease. However, serious adverse effects induced by CARs, especially CRS, GvHD, and immune effector cell-associated neurotoxicity syndrome (ICANS), may occur, and in those cases patients need to be intensively treated during hospitalization [165]. This would significantly increase the cost of CAR-T cell therapy and greatly hamper the clinical transition of CAR-T cell therapy [166]. In comparison to CAR-T, CAR-NK therapy can avoid these problems to a certain extent. For allogeneic CAR-NK cells, it has higher possibility of recognizing tumors through an MHC-independent manner with lower incidence of CRS [142]. Meanwhile, allogeneic NK cells that express KIRs, which do not recognize HLA-I molecules on patient cells, are able to prevent GvHD [167]. Thus, various sources of allogeneic NK cells, such as NK-92 cell line, umbilical cord blood, human embryonic stem cells and iPSCs-derived NK cells expand the pool of NK cell storage, which can make the CAR-NK products rapidly generated as needed without limitations of single source [168]. In addition, NK cells but not T cells can be administered in multiple doses as ‘off-the-shelf’ products because they directly kill tumor cells in the absence of MHC-dependent antigen presentation, which can be transfused to different patients without the need for HLA matching (Fig. 4) [169].

CAR-NK therapy, however, faces several technical challenges, including in vitro cell expansion, gene engineering, and cryopreservation of CAR-NK cells [170]. So far, two main methods to effectively expand NK cells are developed for clinical use. One of them is a feeder cell-based approach. NK cells co-cultured with a leukemia feeder cell line K562 with membrane binding IL-21 and 4-1BBL showed a mean 47,567-fold expansion in vitro [171]. Alternatively, CIML NK cell without feeder cell stimulation is a reliable source for NK cell therapy. In a phase I trial, CIML NK cells survived and comprised more than 90% of blood NK cells at day 7, with an average of (419 ± 166)-fold increase compared with day 1 counts in vivo [29]. The gene delivery technologies, such as murine retroviral and HIV-derived lentiviral vectors, facilitated the studies of CAR related products [172]. This sort of technology has been used to confer human specific CARs to T cells with high-efficiency and stability, and some of these CAR-T cells have been tested in clinical phase I/II studies [173]. However, due to natural resistance of NK cells to the viral infection, the efficiency of viral transduction is relatively low in NK cells [174]. Multiple rounds of transduction plus adjuvants were required to acquire acceptable numbers of CAR-NK cells [116, 175]. Addition of chemical reagents, such as TBK1/IKKε inhibitor BX-795, MRT67303 or amlexanox, enabled an improved transduction of primary NK cells by VSV-G lentivirus from 4.49% to 34.22% [175]. In addition, several studies have shown mRNA electroporation can be used for gene delivery. For example, electroporation of anti-CD19-BB-z mRNA into NK cells resulted in a good efficiency of ~ 52% but only transient gene expression was attained [176].

With the development of immune cell-delivering nanosystem, such as cell surface engineering, nano-bioengager and nanocarriers, encouraging outcomes have been achieved in preclinical experiments [177, 178]. For example, a non-viral lipid nanoparticle-based delivery system that encapsulated small interfering RNAs to silence the key intrinsic inhibitory NK cell molecules was designed. The nanoparticles targeted NK cells in vivo, silenced inhibitory checkpoint signaling molecules, and unleashed NK cell activity to eliminate tumors [178]. Whether these nanosystems could work in strict clinical conditions needs further verification. Besides, iPSCs provide a feasible way to prepare CAR-NK cells from stem cells without direct manipulation of NK cells [118]. It improves the potency of genetic modifications of CARs and enhances expansion and persistence of NK cells [120]. The iPSCs are genetically engineerable to express a CAR, which can differentiate into a homogeneous population of CAR-NK cells. Thus, iPSC-derived CAR-NK cells could be used as a standard and “off-the-shelf” product for anti-tumor immunotherapy while technical efforts are still wanted to lower the cost [179, 180].

Cryopreservation is a critical step in the supply of CAR-NKs as the “off-the-shelf” products. Originally, NK cells are known for their vulnerability to cryopreservation [181]. 10% dimethyl sulfoxide (DMSO) plus 90% FBS is a traditional cryopreservation media, but viability and recovery of thawed CAR-NK cells is only ~ 50% after one month of cryopreservation [182] and only 15% after 5 years of cryopreservation [183]. A DMSO-free cryopreservation medium named IF-M, which is formulated using human serum albumin and glycerol as base components, has recently been developed [182]. The viability, phenotype, and function of CAR-NK cells were evaluated after short-term (90 days) and long-term (1 year) cryopreservation to assess the effectiveness of IF-M. Short-term cryopreservation revealed that the IF-M group outperformed the DMSO group, with 18.85% higher viability and 23.16% higher recovery, respectively. For long-term cryopreservation, there was no significant difference observed between IF-M and DMSO groups in terms of cell viabilities. However, after 1 year of cryopreservation, the cytotoxicity of CAR-NK cells cryopreserved in IF-M was significantly superior to that in DMSO. The CD107a expression intensity of CAR-NK cells in IF-M group was significantly higher than that in DMSO group [182]. Other approaches, such as cryopreservation of NK cells pre-complexed with innate cell engagers [184], and optimization of cryopreservation employing different cooling rates, types of DMSO-containing and DMSO-free cryoprotectants, and cell densities [185], need to be further explored to improve cryopreservation techniques, so widening clinical application of off-the-shelf CAR-NK cells.

### Challenges in adoptive T cell and NK cell-based cancer therapies

A growing number of clinical trials have shown that adoptive T and NK cell therapies are effective against hematologic cancers, but they had limited response from patients with solid tumors. Besides the challenges in clinical procedures as above mentioned, potential limiting factors of infused cells in patients, including restricted infiltration to the tumor sites, lessened activity, suboptimal persistence and antigen escape, deserve research attention (Table 3) [109, 186].

**Table 3:** Potential strategies to overcome current clinical challenges associated with CAR related cell therapies

Issues	Potential strategies
Insufficient tumor infiltration	Local infusion [187, 188]
	Incorporation of chemokine receptors to CAR constructs [191, 210]
	Degradation of ECM to enhance penetration [193, 211]
Immunosuppressive microenvironment	Conversion of inhibitory signals to immunostimulatory signals [196]
	Combination with ICIs to block suppressive signals [212]
Suboptimal persistence	Usage of fully humanized scFv [213]
	Engineering active domain to CAR constructions [172]
	Memory-like cells or stem cell [203, 214]
	Cytokine stimulation [113, 202]
Antigen escape	Target multiple antigens (bispecific or multi-specific CARs) [205, 206]

ECM: extracellular matrix; ICIs: immune checkpoint inhibitors; scFv: variable fragments of signal chain

Infrequent infiltration of infused cells to the core of solid tumors is a vital factor of therapeutic failure due to incompetent killing of cancer cells. Intracranial infusion of CAR-T cells regressed intracranial and spinal tumors, along with the corresponding increases in levels of cytokines and immune cells in the cerebrospinal fluid [187]. Similarly, local delivery of NK cells to hepatic tumors improved therapeutic effects in rodent models of HCC and patients with HCC (KCT0003973) [188, 189]. Lesion location infusion may be more accessible to recruit CAR related cells from circulatory system and avoid on-target off-tumor toxicity than intravenous infusion. However, this strategy may be not suitable for cancers with multiple metastases. Immune cells can be navigated to tumor sites through their chemokine receptors binding to chemokines secreted by tumor cells. Incorporation of chemokine receptors, such as CC-chemokine receptor 4 to CAR-T cells, enhanced CAR-T cell migration to tumors in a mouse xenograft model of Hodgkin lymphoma [190]. Infusion of NK cells expressing high levels of the CXCR2 chemokine receptor resulted in an increased influx of transferred NK cells into renal cell carcinoma and thereby improved clinical outcome of patients [191]. Irrepressible growth and dense extracellular matrix (ECM) of tumor spheres can form physical barrier to impede immune cell homing. Overexpression of heparanase on CAR-T cells could degrade heparan sulfate in the ECM and help T cells trafficking into stroma-rich solid tumors [192]. Human NK cells that expressed low levels of heparanase induced their invasion into tumor and the ensuing tumor suppression [193]. However, heparan sulfate serves as a barrier to tumor invasion and metastasis. This should be taken into account when systemically treating patients with heparanase expressed CAR cells, since the potential adverse effect on ECM degradation might also lead to tumor dissemination [194].

As previously mentioned, immunosuppressive TME impeded both NK cell and T cell activities [195]. Thus, shattering TME might restore the activity of CAR related cells. To convert the suppressive signal induced by TGF-β to an activating signal, NK-92 cells were modified to express a chimeric receptor with TGF-β type II receptor extracellular and transmembrane domains and the intracellular domain of NK cell-activating receptor NKG2D (TN chimeric receptor). NK-92 cells expressing TN receptors were resistant to TGF-β-induced suppressive signaling and inhibited tumor growth in a hepatocellular carcinoma xenograft tumor model [196]. Therefore, TN chimeric receptors can be a novel strategy to augment antitumor efficacy in adoptive cellular therapy. Moreover, PD-1/PD-L1 interaction induced an exhausted phenotype of T cells or NK cells. Blockade of PD-1 signal could partially reverse the function of these exhausted cells with increased proliferation, survival and cytotoxicity [197]. This also worked in adoptive cell therapies to circumvent the immunosuppressive nature of cancers [198].

In vivo persistence of CARs is of great importance for the effective treatment of malignancies. To address this issue, fully humanized scFv specific signaling domains in CARs or sorted memory/stem cells of T cells can prolong the life cycle of CAR-T cells in vivo [199]. For example, CAR-T cells incorporated with CD137 domains survived at least 6 months in mice bearing human ALL xenograft [172]. This may have significant implications for tumor eradication because survival of CAR-T cells for at least a week was required for tumor eradication in a prostate cancer xenograft mouse model [200]. In contrast, NK cells are typically short-lived and lack in vivo persistence in the absence of cytokine support [201]. Exogenous cytokine or cytokine receptor agonist administration or NK cell transgenes with cytokines can be adopted to improve persistence and activation of NK cells after infusion [113, 202]. In addition, CIML NK cells with enhanced persistence and function secreted the amplified IFN-γ for up to 3 weeks after adoptive transfer to Rag 1^−/−^ mice, more durable than NK cells pre-incubated only with IL-15 [203].

Target antigen loss or downregulation is an important form of antigen escape, a major mechanism for tumor relapse in CAR-related therapy [204]. Despite CAR-T cells with single target of CD19 are effective for relapsed and refractory B cell malignancies, relapse with CD19 negative cells remains a technical challenge. In a first-in-human trial of bispecific anti-CD20/anti-CD19 CAR-T cells for relapsed and refractory B cell malignancies (NCT03019055), loss of the CD19 antigen was not seen in patients who relapsed or experienced treatment failure [205]. In addition, B-cell maturation antigen (BCMA) and CS1 bispecific CAR-T cells exhibited superior activity to conventional CAR-T cell that only targeted BCMA in heterogeneous multiple myeloma [206]. Thus, bispecific CARs or other multi-targeting drugs may improve NK and T cell responses by alleviating antigen escape and relapse.

## Conclusions

NK cells are MHC-independent cytotoxic lymphocytes in the innate immune system. When encountering tumor cells, they activate and respond rapidly by releasing cytotoxic granules to eliminate them. With merits including abundant sources and few side effects, NK cell-based cancer therapy emerges as a prospective treatment in experimental and clinical medicine. NK cell directed immunotherapy has demonstrated therapeutic safety and effectiveness for patients with advanced hematologic malignancies, while preclinical animal studies also showed encouraging results in NK cell treatment of solid tumors.

Assuredly, strategies to maximize the therapeutic effectiveness of NK cells deserve further exploration. First, we need to understand how the immunosuppressive TME including inhibitory molecules (such as TGF-β and IDO) and suppressive cells (predominantly Treg and MDSCs) affects the antitumor activity of NK cells. Ablating these barriers is a challenge for the implementation of NK cell-based therapy. Second, the deficiency of growth factors which support the proliferation and survival of NK cells in the TME is another factor that limits the antitumor efficiency of NK cells. Future therapies need to apply growth factors (such as IL-15) to extend the persistence of NK cells in vivo. Third, to increase tumor infiltration of NK cells, gene modified NK cells through induction of chemokine receptor or CARs and their combination therapies with antibodies, engagers and proteins, need to be carefully selected in future clinical trials.

Last but not the least, for the large-scale generation of NK cell products, many studies have taken advantages of MLNK, NK cells derived from HPCs or iPSCs for clinical trials. Also, manipulation of NK cells originating from iPSCs may decrease the difficulties of gene modification and increase specificity and homogeneity of NK cells for individualized treatment. We expect that these new modalities will overcome issues in NK cell-based therapies and provide a universal off-the-shelf product for the treatment of patients with cancer. Overall, based on existing strategies and approaches as discussed in this review, further efforts addressing NK cell activation and expansion, ex vivo manipulation, in vivo persistency and specificity, and effective availability to solid tumors, are urgently and largely needed.

## Data Availability

Not applicable.

## Abbreviations

ADCC: Antibody-dependent cellular cytotoxicity
ALL: Acute lymphocytic leukemia
AML: Acute myeloid leukemia
BCMA: B-cell maturation antigen
BiKE: Bispecific killer engager
CAR: Chimeric antigen receptor
CIM L: Cytokine induced memory-like
CISH: Cytokine-inducible SH2-containing protein
cNK: conventional natural killer cells
CRS: Cytokine release syndrome
DC: Dendritic cell
DNAM1: DNAX accessory molecule 1
DMSO: Dimethyl sulfoxide
ECM: Extracellular matrix
EVs: Extracellular vesicles
FasL: Fas ligand
GM-CSF: Granulocyte–macrophage colony-stimulating factor
GMP: Good manufacturing practice
GvHD: Graft-versus-host disease
GBM: Glioblastoma
HCC: Hepatocellular carcinoma
HER2: Human epidermal growth factor receptor 2
HLA: Human leukocyte antigen
HPCs: Hematopoietic progenitor cells
HSCT: Hematopoietic stem cell transplantation
ILCs: Innate lymphoid cells
IFN-γ: Interferon-γ
IL-15: Interleukin-15
IL-15R: IL-15 receptor complex
IDO: Indoleamine 2,3-dioxygenase
IC: Immunocytokine
ICANS: Immune effector cell-associated neurotoxicity syndrome
iPSCs: Induced pluripotent stem cells
KIRs: Killer cell immunoglobulin-like receptors
mAbs: monoclonal antibodies
MHC: Major histocompatibility complex
MICA/B: MHC class I chain-related A and B genes
MDS: Myelodysplastic syndrome
MDSCs: Myeloid-derived suppressor cells
MLNK: Memory-like Natural killer cells
MM: Multiple myeloma
NK: Natural killer
NKG2D: NK group 2 member D
MSLN: Mesothelin
NCR: Natural cytotoxicity receptor
NSCLC: Non-small cell lung cancer
PD-1: Programmed cell death-1
PD-L1: Programmed death-ligand 1
PGE2: Prostaglandin E2
PDAC: Pancreatic ductal adenocarcinoma
PFS: Progression-free survival
scFv: Variable fragments of single chain
SCLC: Small cell lung cancer
TA: Tumor antigen
TAMs: Tumor-associated macrophages
TCR: T cell receptor
TNF-α: Tumor necrosis factor alpha
TGF-β: Transforming growth factor beta
TME: Tumor microenvironment
TRAIL: TNF-related apoptosis inducing ligand
Treg cells: Regulatory T cells
trNK: tissue-resident NK
TriKE: Trispecific killer engager
ULBP: UL16 binding protein
VB6: Vitamin B6
